# Characterization of Epstein Barr Virus Latency Pattern in Argentine Breast Carcinoma

**DOI:** 10.1371/journal.pone.0013603

**Published:** 2010-10-22

**Authors:** Mario A. Lorenzetti, Elena De Matteo, Hugo Gass, Paula Martinez Vazquez, Julia Lara, Pedro Gonzalez, María Victoria Preciado, Paola A. Chabay

**Affiliations:** 1 Molecular Biology Laboratory, Pathology Division, Ricardo Gutiérrez Children Hospital, Ciudad de Buenos Aires, Argentina; 2 Pathology Division, Ricardo Gutiérrez Children Hospital, Buenos Aires, Argentina; 3 Gynecology Division, Magdalena Villegas de Martinez Hospital, Tigre, Provincia de Buenos Aires, Argentina; 4 Pathology Division, Magdalena Villegas de Martinez Hospital, Tigre, Provincia de Buenos Aires, Argentina; Duke University, United States of America

## Abstract

**Introduction:**

Epstein-Barr virus (EBV)-associated tumors show different expression patterns of latency genes. Since in breast carcinoma this pattern is not yet fully described, our aim was to characterize EBV latency pattern in our EBV positive breast carcinoma series.

**Methods:**

The study was conducted on 71 biopsies of breast carcinoma and in 48 non-neoplastic breast controls. EBNA1, LMP2A and LMP1 expression was assessed by immunohistochemistry with monoclonal antibodies, while viral genomic DNA and EBERs RNA transcripts expression was performed by *in situ* hybridization. EBV presence was confirmed by PCR.

**Results:**

EBV genomic DNA and EBNA1 expression were detected in 31% (22/71) of patients specifically restricted to tumor epithelial cells in breast carcinoma while all breast control samples were negative for both viral DNA and EBNA1 protein. LMP2A was detected in 73% of EBNA1 positive cases, none of which expressed either LMP1 protein or EBERs transcripts.

**Conclusions:**

These findings suggest that EBV expression pattern in the studied biopsies could be different from those previously observed in breast carcinoma cell lines and lead us to suggest a new, EBNA1, LMP2A positive and LMP1 and EBERs negative latency profile in breast carcinoma in our population.

## Introduction

Epstein-Barr virus (EBV) is a ubiquitous human γ-herpes virus (genus *lymphocryptovirus*) that has been linked to a variety of lymphoid and epithelial malignancies, such as Burkitt (BL), Hodgkin (HL) and NK/T lymphomas, nasopharyngeal (NPC) and gastric carcinoma (GC).[1] Given that the list of EBV-related malignancies continues to increase, the World Health Organization classified EBV as a carcinogenic agent in 1997.[2]

All herpes viruses display two phases in their infective cycle that together describe persistent infection; these are latency and lytic replication.[3] In EBV-associated tumors, the virus establishes a latent infection, which is characterized by the limited expression of a subset of viral latent genes. The particular expression pattern of different latent genes defines three latency programs specific to each tumor type. Only EBV-encoded nuclear antigen 1 (EBNA1) is essential for the persistence and replication of the viral genome and is consistently expressed in all types of latencies.[4] EBV associated cancers vary markedly in viral prevalence and in patterns of latent gene expression (Table 1) suggesting that EBV may affect cell growth in several ways.[5] Latency III pattern express both EBERs and BARTs transcripts and all the EBV latent proteins: the 6 nuclear antigens (EBNA1, EBNA2, EBNA3A, EBNA3B, EBNA3C and EBNA-LP) and three membrane proteins (LMP1, LMP2A and LMP2B), and is characteristic for lymphoblastoid cell lines and post-transplant lymphoproliferative disease. Latency II pattern express the EBERs, BARTs transcripts, EBNA1 protein and the latent membrane proteins (LMP1, LMP2A and LMP2B), and is associated to HL and NPC. Finally, latency I is found in BL and GC and is only restricted to the expression of both transcripts and EBNA1 protein. While B cells have the potential to support any of these three types of latent infection, non-B cells generally display either a Latency I or Latency II type of infection.[6]

**Table 1:** EBV gene latency programs.

Latency program	EBV genes expressed						Associated Malignancy
	EBERs/BARTs	EBNA1	LMP1	LMP2A	EBNA2	EBNA3s/EBNA-LP	
0	+	N.D.	-	+	-	-	Peripheral blood memory B cells
I	+	+	-	-	-	-	BL, PEL, GC
II	+	+	+	+	-	-	HL, NPC
III	+	+	+	+	+	+	IM, PTLD
Here described	-	+	-	+/−	N.D.*	N.D.*	Breast carcinoma

N.D.: not determined; BL: Burkitt lymphoma; PEL: Primary effusion lymphoma; PTLD: post-transplant lymphoproliferative disorders; GC: gastric carcinoma; HL: Hodgkin lymphoma; NPC: nasopharyngeal carcinoma; IM: infectious mononucleosis.

*: not determined due to unavailability of commercial antibodies for formalin fixed, paraffin embedded tissue sections.

Breast carcinoma is among the most frequent female malignancies worldwide.[7] Even though there are well-known risk factors associated with it, they only seem to explain about half of the cases.[8], [9] As the etiology of this pathology is poorly understood, novel routes of disease pathogenesis are to be considered. Presently, a large number of infectious agents have been identified which either cause or contribute to specific human cancers, and particularly many studies have suggested that some types of virus might be involved in the pathogenesis of breast cancer.[10] Several laboratories have reported EBV association in a subset of breast tumors, while others have disclosed negative results.[11], [12], [13], [14], [15] It has also been reported that breast cancer cells are heterogeneous in terms of EBV genome content and distribution and this raises the possibility that even though EBV might have no etiological role, it can still contribute to tumor development.[16] Moreover, it has been suggested that EBV could confer breast cancer cells *in vitro* with resistance to chemotherapeutic drugs by means of over expression of a multidrug resistance gene.[17]

Even though EBV latency pattern is extensively characterized in EBV-associated lymphomas, and also in NPC and GC, the latency pattern in breast carcinoma is not yet fully described. A better understanding of EBV expression pattern in this malignancy could ultimately result in novel EBV-targeted therapy which could be applied to patients with EBV positive tumors in addition to conventional chemotherapy. Therefore, our aim was to evaluate EBV latency pattern in this EBV positive breast carcinoma series. To the best of our knowledge this is the first work to describe EBV latency pattern, with LMP2A expression in breast carcinoma.

## Methods

### Ethics statement

This study has the approval of the Institutional Review Board and the Ethics Board of both M. Villegas de Martinez Hospital and Ricardo Gutierrez Children Hospital and is also in accordance with the Helsinki Declaration of 1975, as revised in 1983. A written informed consent was obtained from every patient after the nature of the procedure had been fully explained.

### 1. Patients and samples

The study was conducted on 71 biopsies of breast carcinoma (59 patients previously reported [13]), collected without any preselection criteria from the pathological archives of the Pathology Service of M. Villegas de Martinez Hospital. Tumors, which were typed according to the American Joint Committee on Cancer [18], included 7 invasive lobular carcinomas and 64 invasive ductal carcinomas. Patients' age ranged from 35 to 96 years (median age, 65 years). As controls we studied 17 biopsies of fibroadenomas, 9 of benign epithelial proliferation, 4 of atypical ductal hyperplasia and 10 of usual ductal hyperplasia and 8 normal breast tissues of female patients.

### 2. PCR

DNA was extracted from tumor fixed biopsies using QIAamp DNA Mini Kit (QIAGEN GmbH, Hilden, Germany) following manufacturer's instructions. PCR against EBERs DNA was performed as previously described.[19]

### 3. Immunohistochemistry (IHC)

Formalin-fixed, paraffin embedded tissue sections were assayed for EBV latent membrane protein1 (LMP1) (clone CS1-4, mouse, Dako, Carpinteria, USA), EBV nuclear antigen1 (EBNA1) (clones 2B4 and 1H4, rat) and latent membrane protein2A (LMP2A) (clone 4E11,rat) (all 3 clones kind gift from Dr. Elisabeth Kremmer [19]) immunohistochemical expression with monoclonal antibodies (mAb). For LMP1 mAb, antigen unmasking with citrate buffer pH 6 in microwave oven (533 watts) for five minutes was followed by endogenous peroxidase blockade with 3% H_2_O_2_ for 15 minutes at room temperature. Tris-HCl 0,05 M 1% bovine albumin pH 7.6 was used for nonspecific site blockade. Tissue sections were incubated with a 1/50 dilution of LMP1 for 90 minutes at room temperature. A streptavidin-biotin-peroxidase complex detection system (LSAB, Dako, Carpinteria, USA) was used for amplification and detection. Immunohistochemistry (IHC) for both clones of EBNA1 and for LMP2A was performed with Tris-EDTA 10 mM/1 mM pH 9 unmasking for 5 minutes in microwave oven (533 watts). Endogenous peroxidase was blocked and nonspecific site blockade was performed as for LMP1 antibody. Both EBNA1 clones were incubated at a 1/50 dilution for 1 hour at room temperature. A peroxidase-linked anti-rat antibody (Whole molecule A5795) (Sigma, Missouri, USA) was used for amplification and commercial DAB kit system for detection (Dako, Carpinteria, USA).

Morover, double staining IHC with LMP2A (clone 4E11) and cytokeratin 7 (CK7) (clone OV-TL 12/30, mouse) (Dako, Carpinteria, USA) antibodies was performed. LMP2a was stained as described above followed by staining with CK7. Briefly, LMP2A stained samples were incubated for 30 minutes at room temperature with CK7 and followed by 30 minutes incubation with a biotinylated-anti-mouse antibody at room temperature (Dako, Carpinteria, USA). A streptavidin-biotin-alcaline phosphatase complex detection system was used for amplification and detection (Vector blue AP substrate kit III, Vector Laboratories, Burlingame, USA).

### 4. EBERs *In situ* hybridization

EBERs RNA *In situ* hybridization (EBERs ISH) to detect EBERs transcripts was performed on paraffin sections according to the manufacturer's instructions using a PNA ISH Detection Kit and EBERs peptide nucleic acid probe labeled with FITC, followed by an alcaline phosfatase linked antibody anti-FITC (Dako, Carpinteria, USA).

### 5. BamH1W DNA *In situ* hybridization

BamH1W DNA *In situ* hybridization (BamH1W DNA ISH) to detect EBV DNA genome was performed with a commercial kit according to manufacturer's instructions using a biotin labeled DNA probe followed by an alcaline phosfatase linked antibody anti-biotin (PanPath, Budel, The Netherlands).

As positive control for EBNA1, LMP1, LMP2A, EBERs RNA ISH BamH1W DNA ISH, B95.8 cells were spined down for 10 minutes at 1200 rpm. Pelleted cells were clotted with two volumes of a 90% ethanol:40%formalin:acetic acid (80∶15∶5) solution during 24 hours and then processed for histology and slides preparation as for tissue. For LMP1 and EBERs RNA ISH we also included an EBV positive Hodgkin lymphoma case as a positive control.

A well characterized EBV negative Hodgkin lymphoma from Ricardo Gutiérrez Children Hospital was used as negative control in each staining procedure.

### 6. Statistical analysis

Statistical analysis was performed using GraphPad InStat software, version 3.05 (Graphpad, San Diego, United States). For the univariate analysis, Chi square test was used to assess the association between categorical variables. All tests were two-sided, and a P value of less than 0.05 was considered statistically significant.

## Results

**Figure 1:**
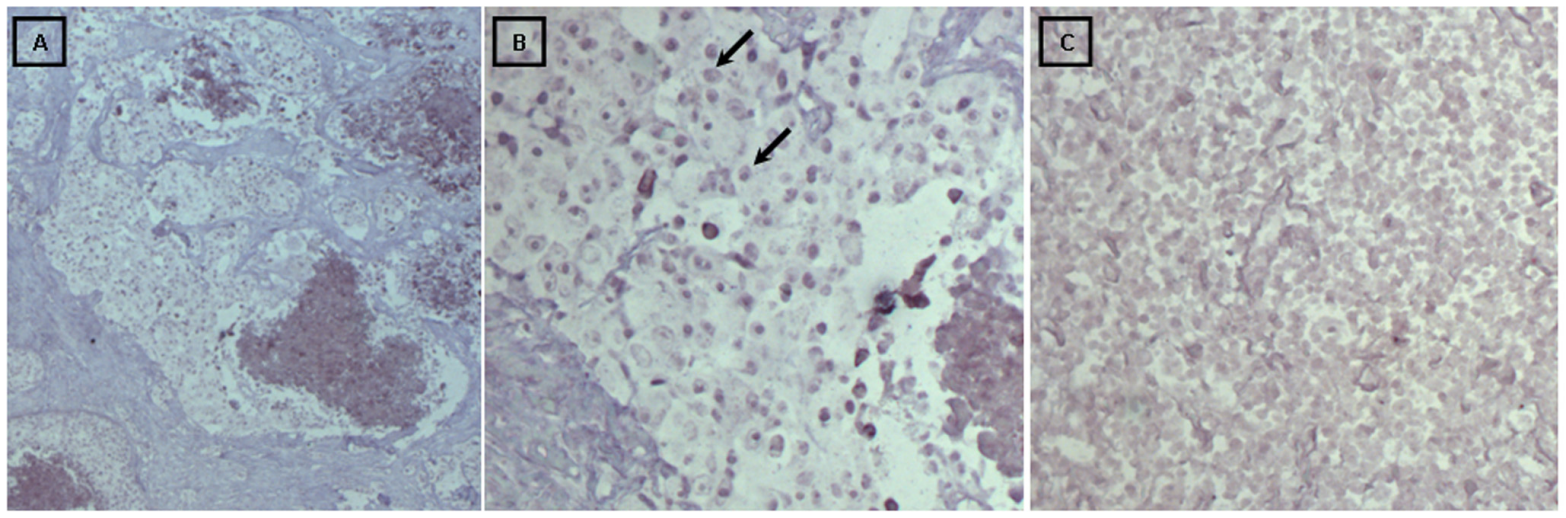
BamH1W DNA *in situ* hybridization in ductal breast carcinoma. Positive BamH1W DNA *in situ* hybridization restricted to nucleus of epithelial tumor cells. **A**) Panoramic view of invasive tumor and necrotic focus (25X). **B**) EBV DNA positive tumor epithelial cells (black arrows) outside necrotic focus (100X). **C**) EBV DNA negative ductal breast carcinoma (100X).

**Figure 2:**
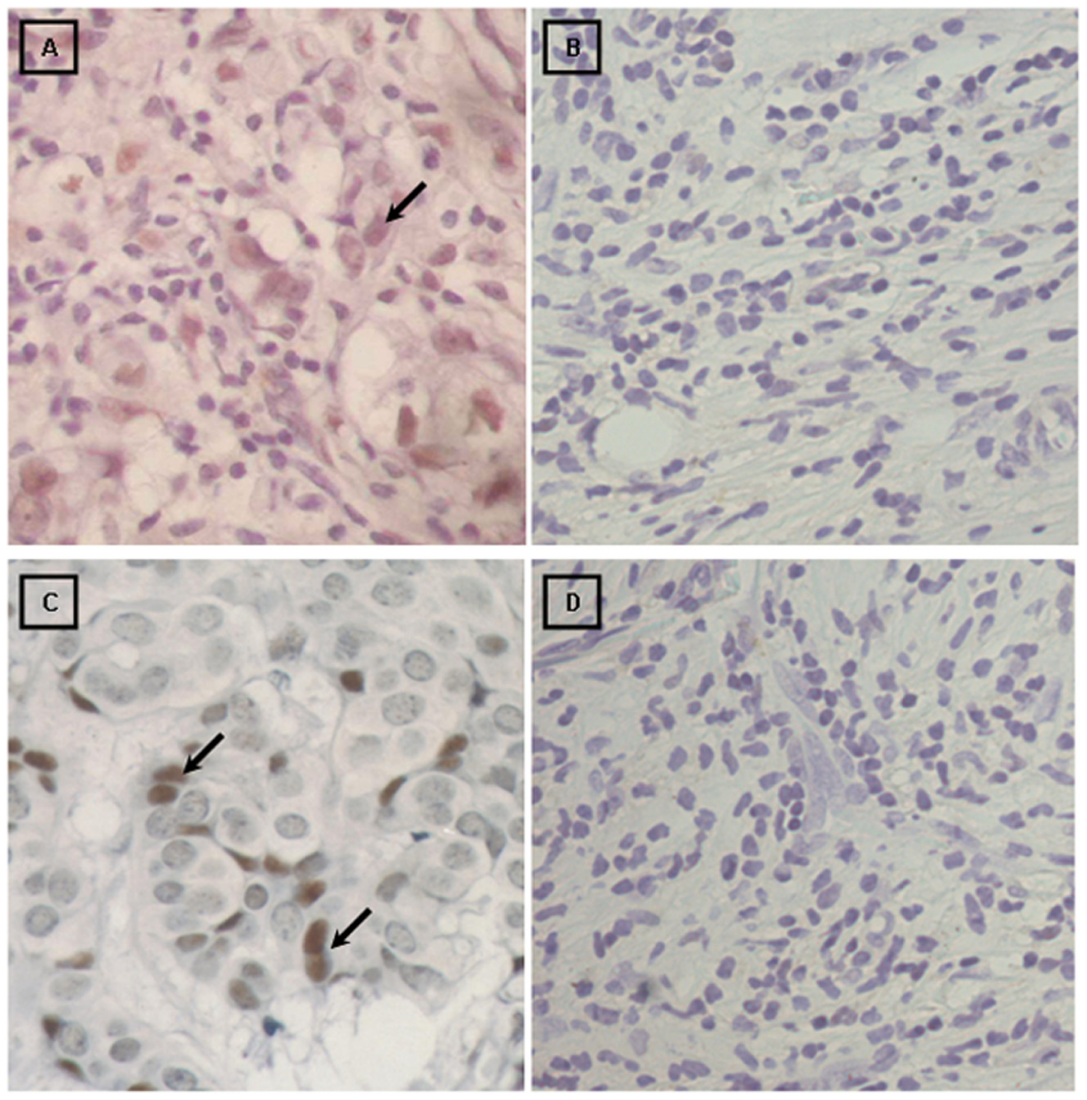
Immunohistochemical detection of EBNA1 (clones 1H4 and 2B4). **A**) Ductal breast carcinoma neoplastic nuclei show positive staining with EBNA1 (clone 1H4) monoclonal antibody, (black arrows), but no infiltrating lymphocyte gave positive signal (400X). **B**) EBNA1 (clone 1H4) negative ductal carcinoma tissue (400X). **C**) EBNA1 (clone 2B4) positive signal in nuclei of neoplastic breast cells (400X). **D**) EBNA1 (clone 2B4) negative ductal carcinoma tissue (400X).

**Figure 3:**
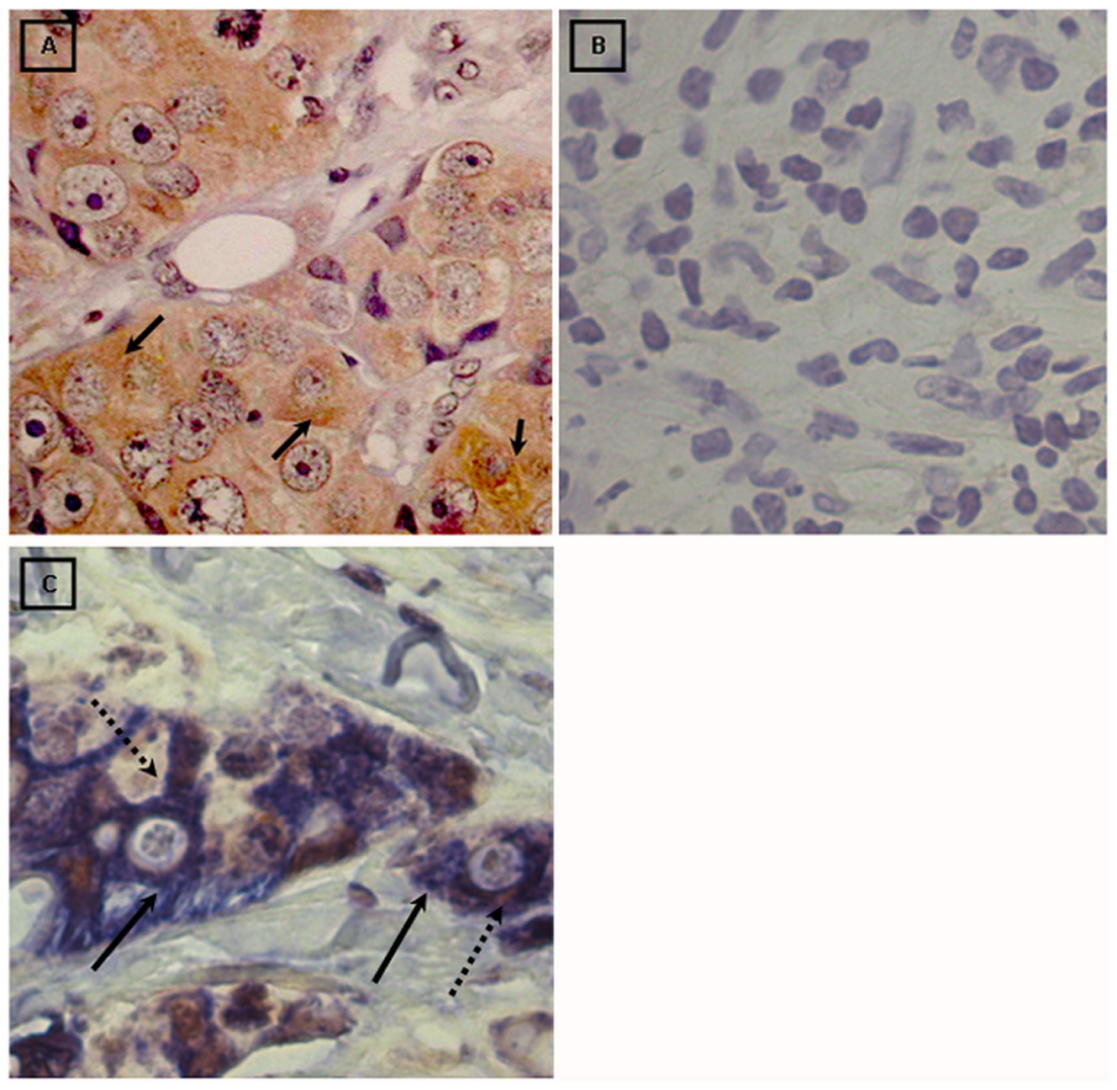
Immunohistochemical detection of LMP2A and double immunohistochemical staining for LMP2A and CK7. **A**) Ductal breast carcinoma with a high number of LMP2A positive cells. Positive signal for LMP2A is restricted to the cytoplasm and membrane of tumor epithelial cells (1000X). **B**) LMP2A negative ductal carcinoma tissue (1000X). **C**) Ductal breast carcinoma. Positive double signal for LMP2A (brown signal, dotted arrow) and CK7 (blue signal, full arrow) restricted to the cytoplasm and membrane of the same tumor epithelial cells (1000X).

Twenty two out of 71 (31%) cases were EBV positive by EBERs specific PCR. To discard the possibility that the positive signal was given by EBV infected infiltrating B-cells within the homogenized tissue, all samples were assayed for viral DNA genome by means of BamH1W DNA ISH and the results matched the 22 EBV EBERs PCR status for all the 71 cases. Nuclear staining corresponding to EBV DNA genome positive hybridization was observed in the nucleus of about 40% of tumor cells in each biopsy, but not in infiltrating B-cells (Figures 1A and B). Breast non-neoplastic controls and the HL case used as EBV negative control were all negative for EBV genome by BamH1W DNA ISH (Figure 1C). EBNA1 expression was analyzed by IHC and all 22 (31%) of 71 EBV DNA positive cases were found to be positive. Granular nuclear staining was observed in tumor epithelial cells in a similar percent as EBV genome hybridization with both clones (1H4 and 2B4), but it was absent in infiltrating B-cells (Figures 2A and C). All non-neoplastic controls as well as the EBV negative HL were negative for both clones of EBNA1 (Figures 2B and D). Although it was reported that the 2B4 clone might not be completely specific for EBV due to cross-reactivity with tumor proteins [20] all our results with the 2B4 clone correlated exactly with the 1H4 clone and also with PCR and EBV DNA genome hybridization results. We then focused on EBV-positive cases by BamH1W DNA ISH to evaluate EBV protein expression and characterize their latency pattern. Therefore, in these cases we analyzed EBERs by EBERs ISH, and LMP1 and LMP2A expression by IHC. LMP2A was detected in 16 (73%) of the 22 EBV DNA positive cases. Positive staining was observed in the cytoplasm and membrane in a variable percentage of tumor epithelial cells but similarly to EBNA1, it was not detected in bystander B-cells (Figure 3A). All control samples were negative for LMP2A (Figure 3B).

**Figure 4:**
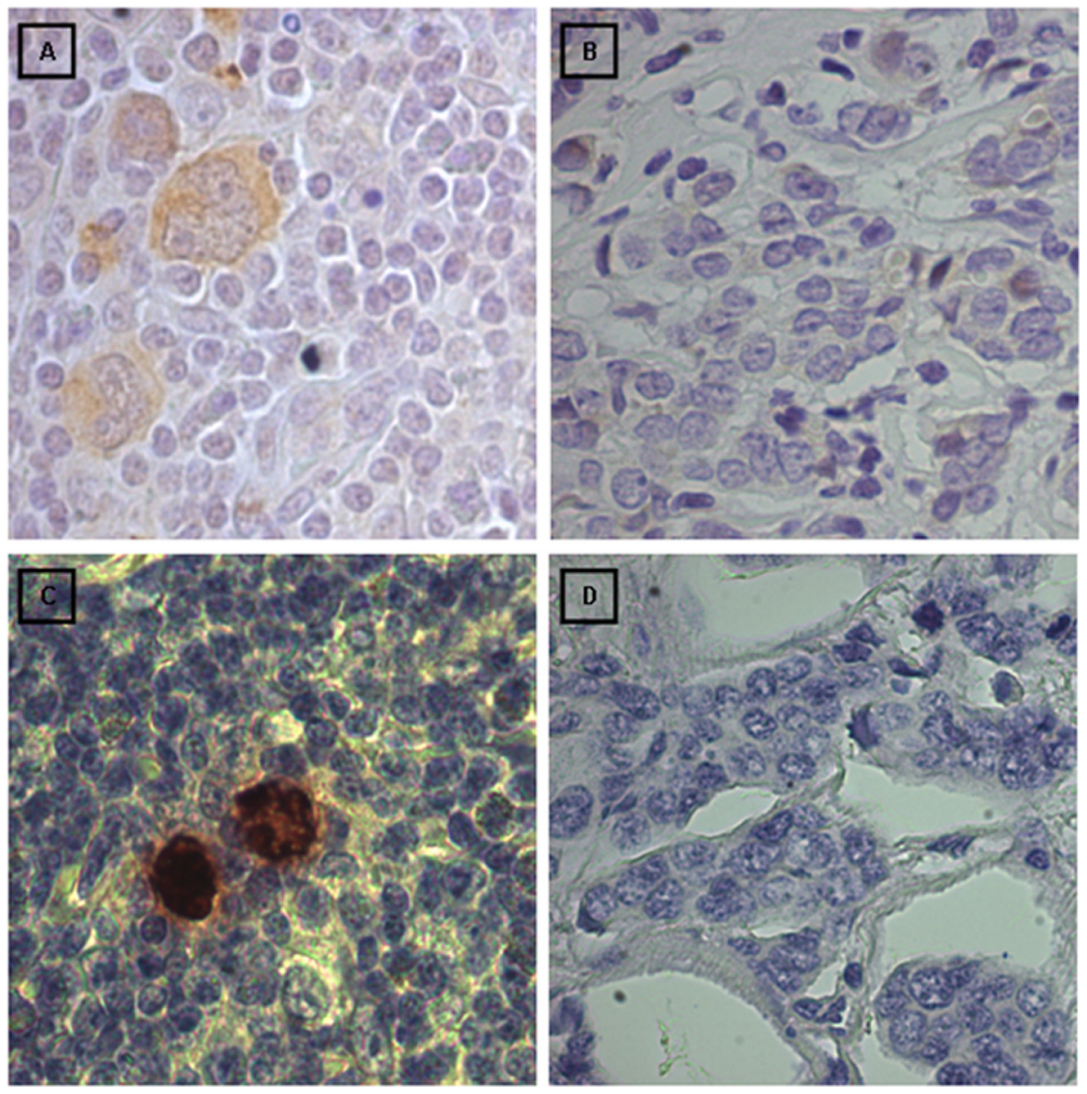
Immunohistochemical controls for LMP1 and EBERs RNA *in situ* hybridization. **A**) HL case: LMP1 positive staining in the membrane of Reed Stenberg cells and HL mononuclear cell (400X). **B**) LMP1 negative ductal breast carcinoma (400X). **C**) EBERs RNA positive staining in HL Reed Stenberg cells (400X). **D**) EBERs RNA negative ductal breast carcinoma (400X).

To further confirm that the LMP2A expressing cells were of epithelial lineage, we performed double staining IHC with LMP2A mAb followed by CK7 mAb. All 16 LMP2A positive samples displayed a double staining pattern in cytoplasm and membrane of EBV infected epithelial neoplastic cells (Figure 3C). None of the EBV positive carcinoma cases or non-neoplastic control samples expressed either LMP1 by IHC or EBERs by EBERs RNA ISH. Figure 4 shows appropriate controls for these assays.

Statistical correlation was assessed between clinical data and three EBV expressed antigen combinations, namely EBNA1-LMP2A-, EBNA1+LMP2A-, EBNA1+LMP2A+. We have previously reported that the presence of EBV was not correlated with clinical outcome. [13] In this work, clinical parameters such as tumor histology, clinical stage, metastasis development and estrogen and progesterone receptors were not statistically associated with any particular EBV antigen pattern (p>0,05 χ^2^ test).

## Discussion

Since 1995, EBV has been described to be associated with a proportion of breast carcinomas by several groups, but others controversially denied this relation. This discrepancy could be in part due to the different techniques used to detect EBV or the molecular target chosen (EBV-derived proteins, -RNA transcripts or -genomic DNA). Another possibility for this discrepancy could be based on the socio-economical background of the patients included in previous studies. It is well established that EBV epidemiology differs between developed and developing regions.[21], [22] While breast tumor biopsies from patients in developed countries in the Northern Hemisphere appear to be EBV negative, a similar percent of association with EBV was described in tumor biopsies from patients in developing countries from the Southern Hemisphere. [11], [12], [13], [14], [15], [23], [24].

Even though PCR is potentially sensitive, it cannot differentiate the source of EBV genome, making it non-suitable for studying tumors with B-lymphocyte infiltrates such as breast carcinoma. On the other hand, both EBERs and BamH1W DNA ISH, as well as IHC for EBV latent proteins allow for the differentiation of positive signaling cells. In this report, detection of EBV genome and EBV-derived antigens was achieved by these three techniques; two of them detected EBV genome, namely EBERs PCR and BamH1W DNA ISH, and the third one detected EBNA1 protein expression with two different clones of monoclonal antibodies (1H4 and 2B4). So far, this is the first report that described EBV genome by BamH1W DNA ISH specifically restricted to breast tumor cells. Although PCR results can be argued against, we found a straight concordance between these and those obtained by BamH1W DNA ISH and IHC for EBNA1. Moreover, in every positive case, whether by BamH1W DNA ISH or IHC, the positive signal was always restricted to tumor cells and never observed in infiltrating lymphocytes.

EBNA1 expression in the absence of both, LMP1 and EBERs, was previously reported in invasive tumor cells.[25] Moreover, an EBERs negative *in situ* hybridization pattern within EBV positive Hodgkin lymphoma cases was also documented. [26] Particularly, EBNA1 expression is essential for viral episome maintenance and replication and it is expressed in all known forms of latency. In the present study we indeed demonstrated the expression of EBNA1 in a subset of breast carcinoma tumor cells by IHC analysis with two different clones of monoclonal antibodies (1H4 and 2B4). Hennard and co-workers [20] reported EBNA1 2B4 mAb cross-reactivity with MAGE-4, a protein expressed in various human cancers. When they compared 2B4 immunostaining against 57B mAb, specific for MAGE-4, in EBV-negative tumors, 3/7 EBV-negative Hodgkin lymphomas were positive with 57B mAb but only 1/7 showed unspecific staining with 2B4 mAb. The fact that only one HL case showed unspecific staining with 2B4 mAb even after staining enhancement with TSA is not sufficient to conclude that this mAb cross-reacts with cellular proteins in this lymphoma. Besides, they used EBERs ISH as criteria for establishing EBV presence in breast carcinoma which seems to be an EBERs RNA negative type of tumor, so the possibility that they analyzed EBV positive breast carcinoma cases cannot be ruled out. Finally, our results with 2B4 mAb and 1H4 mAb are comparable and no staining was observed in non tumor samples which were negative for EBV genome assessed by BamH1W DNA ISH, or in the EBV-negative Hodgkin lymphoma case.

We found EBNA1 expression in 31% of cases which is in agreement with 32% to 52% EBV association previously described in several geographic locations [12], [13], [14], [15], [27], [28], [29] and argues with those who oppose to this association.[23], [24] Together with EBNA1, LMP2A is expressed in several EBV-related tumors which display latency II and latency III expression profile, such as HL and Post-Transplant Lymphoproliferative Disorders, respectively.[4], [30], [31] We found that LMP2A is expressed in 16 (73%) of the 22 EBNA1 positive samples. This finding is opposed to those in previous studies, which analyzed EBV latent gene expression pattern in breast carcinoma and failed to detect expression of EBNA1, LMP1 and LMP2A by IHC as well as EBER by ISH.[23], [24] Additionally, by means of double IHC staining with LMP2A mAb and CK7 mAb we confirmed the epithelial lineage of the EBV positive cells within the tumor.

To the best of our knowledge this is the first study that demonstrates the expression of LMP2A restricted to tumor cells in breast carcinoma biopsies. LMP2A was found to induce the expression of a range of genes that are involved in cell-cycle induction and inhibition of apoptosis, in epithelial and B-cells, by modulating both Ras/PI3-K/Akt and β-Catenin signaling pathways.[32], [33] It was also shown to have repressive effects on the NF-kB pathway, and induce cell migration via interaction with spleen tyrosine kinase (syk).[34], [35] Since it seems that LMP2A has transforming characteristics in epithelial cells, and LMP1 is not expressed in our breast carcinoma series, LMP1 oncogenic capacity could be replaced by LMP2A in our population. Moreover, it can be speculated that in our LMP2A positive cases, LMP1 expression could be down regulated by LMP2A, since it has been reported that LMP2A expression seems to inhibit LMP1 transcription in epithelial cells.[34]

Besides LMP1, we failed to detect EBERs transcripts in all the studied biopsies, which is consistent with previous reports.[23], [36], [37], [38] This data implies that EBERs *in situ* hybridization, which is considered the gold standard method to detect EBV in EBV-associated lymphoid malignancies, is not a suitable method to apply in breast carcinoma.

As previously mentioned, there are three EBV latency patterns described in tumors (Table 1), but EBV latency pattern in breast carcinoma has not been fully characterized. A latency I profile was defined for EBV-infected breast cancer cell lines, since EBNA1 together with EBER1 and BARF0 transcripts were detected, but not EBNA2, LMP1, LMP2A and BZLF1 transcripts.[37] Our results showing LMP2A expression and absence of EBERs transcripts in breast carcinoma biopsies argue against this result, and suggest that EBV expression pattern in the studied biopsies could be different from those observed in *in vitro* cultured cell lines. Controversy regarding EBV latency pattern is not uncommon in EBV-related carcinomas. Even though latency II is defined for EBV-positive NPC, LMP1 expression is detected in about 70% of all cases.[2] Latency II was also originally proposed for gastric carcinoma [39], [40] but recently, the lack of detection of LMP1 in this malignancy better defined EBV latency pattern as latency I.[2], [41], [42] It is important to define EBV protein expression and latency profile in this disease in order to identify viral proteins which interact with cellular factors and deregulate signaling pathways which could at last trigger neoplastic processes.

In summary, this study reinforces EBV association with breast carcinoma in a developing country by a new approach which detected EBV genome specifically localized in breast tumor cells. Furthermore, LMP2A together with EBNA1 expression in absence of EBERs transcripts and LMP1 protein in our series lead us to suggest this new EBV latency profile in breast carcinoma in our population. However, the fact that EBV was detected in only 31% of breast carcinoma biopsies indicates that EBV has no etiological role in breast carcinoma, but it can still contribute to tumor development as a cofactor as previously suggested for EBV positive lymphomas.[16] Further studies, such as the detection of mRNA for all latency genes by real-time PCR, are needed to deeply characterize and redefine EBV latency pattern in EBV-associated breast carcinoma in Argentinean population.
